# Machine learning to detect schedules using spatiotemporal data of behavior: A proof of concept

**DOI:** 10.1002/jeab.70029

**Published:** 2025-06-30

**Authors:** Marc J. Lanovaz, Varsovia Hernandez, Alejandro León

**Affiliations:** ^1^ École de psychoéducation Université de Montréal Canada; ^2^ Centre de recherche de l'Institut universitaire en santé mentale de Montréal Canada; ^3^ Centro de Investigaciones Biomédicas Universidad Veracruzana Mexico

**Keywords:** machine learning, neural network, spatiotemporal data, time‐based schedule

## Abstract

Traditionally, the experimental analysis of behavior has relied on the single discrete response paradigm (e.g., key pecks, lever presses, screen clicks) to identify behavioral patterns. However, the development and availability of new technology allow researchers to move beyond this paradigm and use other features to detect schedules. Thus, our study used spatiotemporal data to compare the accuracy of four machine learning algorithms (i.e., logistic regression, support vector classifiers, random forests, and artificial neural networks) in detecting the presence and the components of time‐based schedules in 12 rats involved in a behavioral experiment. Using spatiotemporal data, the algorithms accurately identified the presence or absence of programmed schedules and correctly differentiated between fixed‐ and variable‐space schedules. That said, our analyses failed to identify an algorithm to discriminate fixed‐time from variable‐time schedules. Furthermore, none of the algorithms performed systematically better than the others. Our findings provide preliminary support for the utility of using spatiotemporal data with machine learning to detect stimulus schedules.

The experimental analysis of behavior typically involves environments that are manipulated by an experimenter to examine the effects of changes in these environments on the behavior of living organisms (Skinner, 1966). These experimental manipulations have contributed to the development of a comprehensive, rigorous, and conceptually systematic natural science of behavior (Catania, 2013). By manipulating environments, researchers have extensively studied the effects of stimulus delivery and behavior‐dependent schedules in both human and nonhuman organisms (Kahng & Iwata, 2005; Schlinger et al., 2008). Traditionally, the identification of these behavioral patterns has relied on the measurement of single discrete responses. This traditional approach, known as the single discrete response paradigm (Henton & Iversen, 1978), relies on behaviors selected for their ease of measurement and applicability such as lever presses for rats, key pecks for pigeons, and screen clicks for humans. However, an organism's behavior is not confined to discrete responses in noncontrived environments. Organisms exhibit spatiotemporal continuity in their behavior, often displaying a diverse range of ecologically relevant behavioral patterns including foraging, water‐seeking, and mating (Hernández et al., 2023; León et al., 2020, 2021).

A substantial body of empirical evidence indicates that spatiotemporal features of behavior are highly sensitive to reinforcement contingencies (Baum & Rachlin, 1969; Hernández et al., 2023; León et al., 2021; Silva & Timberlake, 1997; Timberlake & Lucas, 1985). These features include distance to the reinforcement source, traveled distance, speed, time spent in specific zones, and location entropy. A ranking variable analysis conducted by León et al. (2020) revealed that under temporal reinforcement schedules, spatiotemporal features such as location entropy, traveled distance, and distance to the reinforcement source exhibited greater sensitivity to reinforcement contingencies than discrete responses.

Although previous research has shown that spatial features are sensitive to reinforcement schedules, the next significant step involves identifying the contingencies governing organism behavior using spatiotemporal features. However, using spatiotemporal features to detect schedules presents challenges. Spatiotemporal data provide more naturalistic measures to detect schedules of stimulus delivery in uncontrolled environments, but the amount and characteristics of the data preclude the use of traditional methods of analysis. A promising approach to detecting patterns in these types of data sets is supervised machine learning (see Turgeon & Lanovaz, 2020). In supervised machine learning, the experimenter provides the computer with input data (i.e., features) as well as the output data that it should identify (i.e., labels). The computer then uses an algorithm (i.e., a set of computer instructions) to identify the output using the input data alone. Consequently, it may be possible to train a model to use spatiotemporal data as input to detect the presence of schedules as output.

Several researchers have examined the use of machine learning to predict responding or detect reinforcement histories (e.g., Lusk, 2019; Plessas et al., 2022; Raphan et al., 2019). Notably, Plessas et al. (2022) applied spiking neural networks to identify concurrent ratio schedules in six pigeons. The experimental arrangement included two keys: left and right. The ratio of left : right key pecks following the delivery of reinforcement was used as an input variable to train models to identify the schedules. Their study showed that machine learning could correctly identify the ratio of reinforcement in a session by relying solely on the ratio of key pecks with a high level of accuracy. Their promising results suggest that machine learning may effectively identify reinforcement histories in pigeons. Other researchers have used neural networks to better identify interval timing in both rats and pigeons (see Lusk, 2019; Raphan et al. 2019). Although the results are promising, none of the prior studies used spatiotemporal data to identify the schedules. Using spatial features may more closely approximate the natural behavior of living organisms than using lever presses and key pecks. Thus, our study applied four machine learning algorithms to spatiotemporal data to identify the presence and the components of time‐based schedules in a behavioral experiment conducted with 12 rats. Our main research question was as follows: Can analyzing spatiotemporal data with machine learning detect schedule components in rats?

## METHOD

### Subjects

To train and test our models, we used data that were collected as part of a prior experiment that examined the effects of time‐based schedules and spatial location of water sources on spatiotemporal behavior in rats (León et al., 2023). The subjects of the study were 12 experimentally naïve Wistar rats. Each rat was 3 months old at the beginning of the experiment, housed individually, and under a 23‐hr water restriction with free 30‐min access at the end of each session. Food was freely available in their home cages. Home cages were housed in a colony room with 12‐hr light : 12‐hr dark cycles, with lights on at 7:00 a.m. Sessions were conducted daily, seven days per week between 8 a.m. and 2 p.m. All procedures complied with university regulations for animal use and care and with the Mexican norm NOM‐062‐ZOO‐1999 for Technical Specification for Production, Use and Care of Laboratory Animals.

### Apparatus and measures

#### Apparatus

The experimental chamber was 92 cm wide, 92 cm long, and 33 cm in height. The center of each wall included a liquid dipper (Coulbourn E14‐05) located 2 cm above the grid floor, which allowed access to 0.1 cm^3^ of water for 3 s. Head entry detectors (MED ENV‐254‐CB) identified entries into the four dispensers. The chamber also included a buzzer located in the upper central part of the chamber that presented a tone to signal water availability. The MED PC IV software programmed and recorded water deliveries as well as head entries using a connected computer outside the experimental chamber. A video camera (Topica TP‐505D/3), located 1 m above the chamber, recorded the real‐time location of the subjects in the experimental chamber. Finally, we analyzed the video recordings using Ethovision XT. The software provided records of the rat's movements in *x*, *y* coordinates every 0.2 s.

#### Measures

We used eight different measures derived from discrete responses and movements in the experimental chamber. The first four measures were the total number of head entries into each one of the four water dispensers. The other four measures were *traveled distance*, *standard deviation of speed, location entropy*, and *divergence*.^1^ Traveled distance involved measuring the total distance (cm) that the rat moved in a session. The standard deviation of speed was calculated for each session (consisting of 6,000 speed data points) for each subject. The speed variable represents a frame‐by‐frame calculation (at a resolution of 5 Hz) of the distance traveled per unit of time, as measured by Ethovision XT. The standard deviation was then computed for each session to measure the variability in speed across frames, capturing how consistently the subject's speed varied throughout the session. This process allowed us to assess the fluctuation of speed over time for each subject in their respective sessions under each condition.

An entropy measure was used as an indicator of locomotion pattern variation, enabling us to capture the dynamics of behavior under different reinforcement schedules (León et al., 2021). Higher entropy values indicate greater variation in position. On the other hand, divergence serves as a measure of spatial behavior variation, specifically in the organism's location between consecutive sessions (León et al., 2021). Our analyses computed divergence by comparing the distribution of the organism's locomotion within the chamber across two consecutive sessions.

Location entropy was derived from Shannon entropy. Shannon entropy is a measure associated with discrete random variables that quantifies variability within a distribution. It serves as a continuous, monotonic, and linear indicator of the dissimilarity between elements within the distribution (Carcassi et al., 2021). Formally, given a discrete random variable X, with possible states $\left\{x_{i}\right\}$, each with probability $P\left(x_{i}\right)$, the entropy $H\left(X,P\right)$ is calculated as follows (see Equation 1):

(1) $$H\left(X,P\right)=-\sum P\left(x_{i}\right)\ln\left(P\left(x_{i}\right)\right).$$

The entropy of a discrete random variable is a nonnegative number, $H\left(X,P\right)\geq 0$. To assess location entropy and analyze the variability of position within each session, we used discrete random variables $\left\{x_{i}\right\}$, representing the time spent in each of the predefined 10×10 zones; $P\left(x_{i}\right)$ represents the accumulated (standardized) time spent in each zone.

Finally, divergence, also known as relative entropy, is a measure of the difference between two probability distributions of the organism's location. For discrete probability distributions P and Q defined on the same random variable X, with possible outcomes $\left\{x_{i}\right\}$, the Kullback–Leibler divergence from Q to P is defined as follows (see Equation 2):

(2) $$D_{\mathrm{KL}}\left(P∥Q\right)=\sum_{i}P\left(x_{i}\right)\ln\left(\frac{P\left(x_{i}\right)}{Q\left(x_{i}\right)}\right)$$

The Kullback–Leibler divergence is defined only when, for all x, $Q\left(x\right)=0$ implies $P\left(x\right)=0$. When $P\left(x\right)$ is zero, the contribution of the corresponding term is considered as zero. The Kullback–Leibler divergence $D_{\mathrm{KL}}\left(P∥Q\right)$ can be seen as a measure of how different the distribution of Q is from the distribution P, as it is always nonnegative—$D_{\mathrm{KL}}\left(P∥Q\right)\geq 0$. A crucial point to note is that $D_{\mathrm{KL}}\left(P∥Q\right)$ is zero if and only if P=Q. Put differently, a Kullback–Leibler divergence of 0 indicates that the two distributions are identical. The measure is not symmetric; that is, $D_{\mathrm{KL}}\left(P∥Q\right)\neq D_{\mathrm{KL}}\left(Q∥P\right)$. To analyze the rat's location across consecutive sessions, we used discrete random variables $\left\{x_{i}\right\}$ that represented the time spent in each square region within a 10 × 10 defined zone configuration. The parameter $Q\left(x_{i}\right)$ denotes the accumulated time (standardized) during the first session, whereas $P\left(x_{i}\right)$ represents the accumulated time (standardized) during the second session.

### Procedures

We randomly assigned subjects to one of four groups (i.e., three subjects per group), which were all exposed to two phases in the same order. In the first phase, each rat was subjected to one of the four following schedules for 30 sessions: 30‐s fixed time (FT)–fixed space, 30‐s FT–variable space, 30‐s variable time (VT)–fixed space, and 30‐s VT–variable space (see Table 1). During the FT and VT schedules, water delivery was always signaled with a 3‐s tone immediately prior to delivery. The VT schedules were programmed using the Hoffman–Fleshler constant probability distribution with a list of seven intervals (3, 7, 13, 21, 31, 47, and 88 s; see Fleshler & Hoffman, 1962). During the fixed‐space condition, the water delivery always occurred in the same dispenser, whereas during the variable space condition, water delivery occurred randomly in one of the four different dispensers. In the second phase, the rats no longer had access to water delivery for 10 sessions (no programmed schedule). The duration of each session was 20 min. We used a fixed criterion of 30 sessions, based on prior research in similar conditions, which indicated that stability is typically achieved within that number of sessions (Hernández et al., 2023; León et al., 2021).

**TABLE 1:** Schedule components for each rat.

	Schedule components	
Rat	Space	Time
S1	Fixed	Fixed
S2	Fixed	Fixed
S3	Fixed	Fixed
S4	Fixed	Variable
S5	Fixed	Variable
S6	Fixed	Variable
S7	Variable	Fixed
S8	Variable	Fixed
S9	Variable	Fixed
S10	Variable	Variable
S11	Variable	Variable
S12	Variable	Variable

### Algorithms

As our study was the first to examine the use of machine learning in combination with spatiotemporal data to identify schedule components, which machine learning algorithm would perform best on this type of data was unclear. Therefore, the study involved comparing four algorithms: logistic regression, support vector classifiers, random forests, and artificial neural networks. Table 2 presents a brief description of each algorithm as well as its strengths and weaknesses. A more detailed description of each algorithm can be found below. All our data and code are freely available at https://osf.io/uqhxm/.

**TABLE 2:** Description of the algorithms (Boulesteix et al., 2012; Cervantes et al., 2020; Dreiseitl & Ohno‐Machado, 2002; Tu, 1996).

Algorithm	Brief description	
Logistic regression	The logistic regression is a generalized linear model that relies on the sigmoid or logit function (i.e., S‐shaped) to classify the data.	
	Strengths: Simple to implement Efficient Explainable coefficients	Weakness: Limited to linear boundaries
Support vector classifier	The support vector classifier uses a function that adds a new dimension to data to separate the classes more easily using a (hyper)plane.	
	Strengths: Nonlinear separation of data Deterministic (i.e., always produces the same model)	Weaknesses: Computationally inefficient with large data sets Difficulty in classifying imbalanced sets
Random forest	The random forest procedure involves building multiple decision trees to classify the data; each tree then votes to determine how to classify novel exemplars.	
	Strengths: Nonlinear separation of data Identifying variable importance	Weaknesses: Computationally inefficient with large data sets Loss of information due to discretization of continuous data
Artificial neural network	The artificial neural network transforms the data nonlinearly from one layer to another to model complex patterns in data.	
	Strengths: Identifying of complex nonlinear relationships Modeling of all interaction effects Computationally efficient with large data sets	Weaknesses: Complex to train (many hyperparameters to consider) Risk of overfitting Instability, especially for small data sets Low explainability

#### Logistic regression

Logistic regression^2^ involves separating the data into classes, or categories, using linear boundaries. From a mathematical standpoint, the algorithm identifies weight values that will minimize classification errors produced by the sigmoid, or logit, function (i.e., S‐shaped). In contrast to linear regression, there are no closed‐form solutions to identify these weights, so researchers have developed several optimization algorithms to solve this problem (Goodfellow et al., 2016). The current study used the liblinear solver, which is recommended for small data sets. This solver uses coordinate descent, an algorithm that attempts to find the minimum of a function by iteratively decreasing the error along coordinate directions (Wright, 2015). An L2 regularization term, sometimes referred to as a ridge regression, was applied to mitigate multicollinearity and reduce overfitting. We included logistic regression in the study because it is well known by researchers, simple, and easier to implement than other algorithms are.

#### Support vector classifier

The support vector classifier adds a dimension to the data to facilitate their separation, which may involve a nonlinear boundary. More specifically, the algorithm relies on a kernel, or a mathematical function, to project the data in a higher dimension and then splits the data using a hyperplane designed to maximize the margins around the latter (Witten et al., 2017). The current study used the radial basis function kernel because it produces nonlinear separation of the data. The support vector classifier typically performs well on small data sets, and its mathematical foundations differ considerably from those of the other algorithms, making it relevant to test as part of the current study.

#### Random forest

The random forest procedure involves the creation of a series of decision trees (i.e., a forest). When novel data are presented to the algorithm, they pass through each decision tree, which votes on the assignment of the data to a specific category or class (Breiman, 2001). The sample is then classified in the category that obtained the highest number of votes. To create each tree, the algorithm randomly selects samples and then creates nodes that maximize information gain (i.e., reduces entropy) at each split. In our case, the random forest produced 100 trees that each voted to determine the classification of any given sample. Similar to the support vector classifier, random forest runs efficiently on small samples and its tree‐based method differs considerably from that of the other algorithms in this study, making it an appropriate algorithm to include in the comparison.

#### Artificial neural network

The artificial neural network was the final algorithm that we tested as part of the current study. In contrast to prior studies that used spiking neural networks to train their models (e.g., Plessas et al., 2022), our analyses relied on fully connected (dense) artificial neural networks. Despite the promising nature of spiking neural networks, we selected artificial neural networks because the latter are generally more mature, accessible, and accurate (for a comparison, see Niu et al., 2023). In addition, we did not have sufficient data to tune the parameters of a spiking neural network. In a dense neural network, an algorithm passes the data through a series of layers each of which produces a nonlinear transformation. This transformation involves matrices of initially random numbers that are updated as the model learns. Thus, the error of the model decreases over time until training is completed.

Mathematically, the input, hidden (i.e., middle), and output layers are multiplied by weight matrices to which activation functions are then applied to produce nonlinear transformations, which will eventually result in an output. The algorithm uses this output to compute the gradient of the error and backpropagates this gradient to update the weight matrices. This process is repeated several times (epochs) to reduce the error. In our case, the first hidden layer included five neurons (slightly more than half the number of input neurons; see Heaton, 2015) and the second hidden layer had three neurons. For activation, our algorithm used a ReLU function for the hidden neurons and a sigmoid function for the output. Our network relied on the binary cross‐entropy function to compute the loss (error), and training stopped when the loss had not improved by more than 0.001 for 10 continuous epochs.

### Analyses

#### Features

Each machine learning algorithm uses features as input data to train the models and classify the samples. The features were the eight measures that were extracted by the apparatus: entries into each of the four dispensers, traveled distance, standard deviation of speed, location entropy, and divergence. Prior to being processed by the algorithms, these measures were transformed to *z* scores to address concerns related to saturation and to facilitate the convergence of the artificial neural networks. The features were the same across all analyses.

#### Class labels

In machine learning, class labels represent the output of the algorithm (i.e., the dependent variable in traditional statistics). Our process trained the models to identify three binary class labels: (1) the presence vs. absence of any programmed schedule, (2) fixed‐space vs. variable‐space schedules, and (3) FT vs. VT schedules. When training and testing the models, we excluded the first five sessions of each phase to increase stability in responding. For the fixed‐ vs. variable‐space and the FT vs. VT analyses, the second phase was excluded entirely from our analyses because it did not contain any programmed schedule to differentiate.

Although we trained and tested models to differentiate the individual schedule components (e.g., fixed vs. variable space; FT vs. VT), we could not do the same for the four unique schedules (i.e., multiclass analysis). The problem was that each unique schedule was tested with only three rats (see Table 1). Our cross‐validation required that we leave the target rat out to train the models (see below), so only two rats would be left to classify the behavior of the third rat with the same schedule. This amount of data is insufficient to produce generalizable machine learning models.

#### Cross‐validation

**FIGURE 1:**
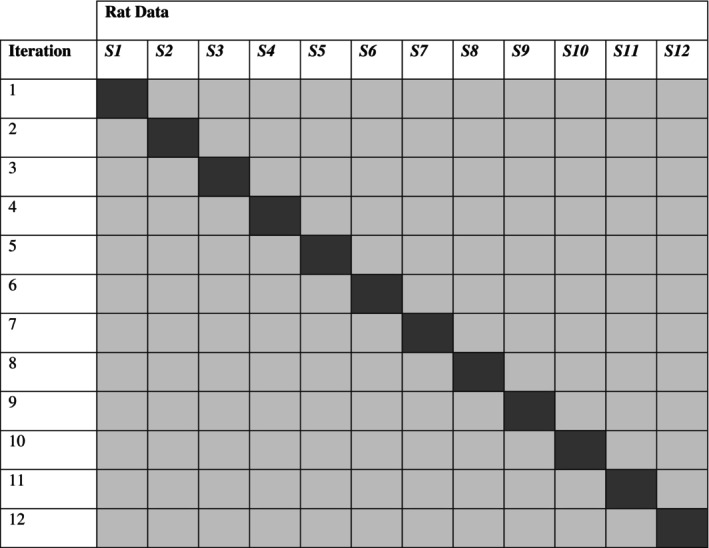
Diagram of the cross‐validation design. The light gray cells identify the training data, and the dark gray cells identify the test data.

One of the main purposes of developing models to detect the presence of a time‐based schedule includes identifying the condition of rats whose data were not used to train the model. In other words, do the models that are trained with some rats generalize to other rats? To address this issue, we used an adapted version of the *k*‐fold cross‐validation method (Yarkoni & Westfall, 2017). That is, each model was trained on 11 rats and tested on the remaining rat (12‐fold cross‐validation). This process was repeated 12 times so that each rat was in the training set 11 times and in the test set once (see Figure 1). Therefore, models were tested only on rats that had not been included in the training, which is necessary to examine the generalizability of the models.

Our cross‐validation procedures meant that one value of the binary class always contained fewer samples than the other. The problem with having an unbalanced training set is that some errors (e.g., false positives) carry more weight than others (e.g., false negatives), skewing the models. To control for this unbalanced training set issue, our code randomly oversampled sessions from the minority class so that our training set contained an equal number of sessions from both classes.

#### Outcomes

For the first class label (i.e., the presence vs. absence of any programmed schedule), our outcome measures included the discriminability index (*d*′), the *c* bias term, sensitivity, and specificity. The discriminability index measures the distance between the signal and the noise in standard deviations, whereas the *c* value represents the bias of the classification. (Stanislaw & Todorov, 1999). Values of *d*′ larger than 0 indicate that the model has more true positives than false positives, whereas values smaller than 0 indicate more false positives than true positives (i.e., it was more likely to classify a pattern as arising from a “schedule” when a schedule was absent than when it was present). A *c* value of 0 identifies models with no specific bias, whereas values greater and smaller than 0 indicate conservative and liberal criterion biases, respectively. Sensitivity, also known as true‐positive rate, involves the proportion of true positives correctly identified by the model. It is calculated by dividing the number of true positives by the sum of true positives and false negatives. Conversely, specificity, or true negative rate, considers the proportion of correctly identified true negatives and involves dividing the number of true negatives by the sum of true negatives and false positives.

For the two other class labels (i.e., variable vs. fixed space and FT vs. VT), the sessions of the test set always had the same value because each rat contacted only one of the schedules. Therefore, it was not possible to compute *c*, sensitivity, and specificity. Thus, our study measured only *d*′ for only these two class labels. We derived these values by computing accuracy and then using a table developed by Hacker and Ratcliff (1979) to identify *d*′ for a two‐alternative forced choice.

#### Comparisons

As indicated previously, a model that produces accurate discrimination should result in *d*′ values larger than 0. To examine whether the models resulted in positive *d*′ values at a statistically significant level, we used one‐sample Wilcoxon signed‐rank tests. Our analyses tested 12 different models (four algorithms for each of the three classes), but the results suffered from familywise error produced by conducting multiple tests. To provide better control over Type I error rate, we applied a Bonferroni correction to our alpha of .05; this resulted in only *p* values equal or lower than .004 being considered significant. To determine whether the algorithms produced models that resulted in statistically different *d*′ values, we also applied a Friedman chi‐square test to each class. Our analyses relied on nonparametric tests because the small sample size could not ensure that our distributions met the assumptions for parametric tests and our data set had outliers. Moreover, we were more concerned with the rank of each algorithm (i.e., which performed better) than with differences in mean performance.

## RESULTS

**FIGURE 2:**
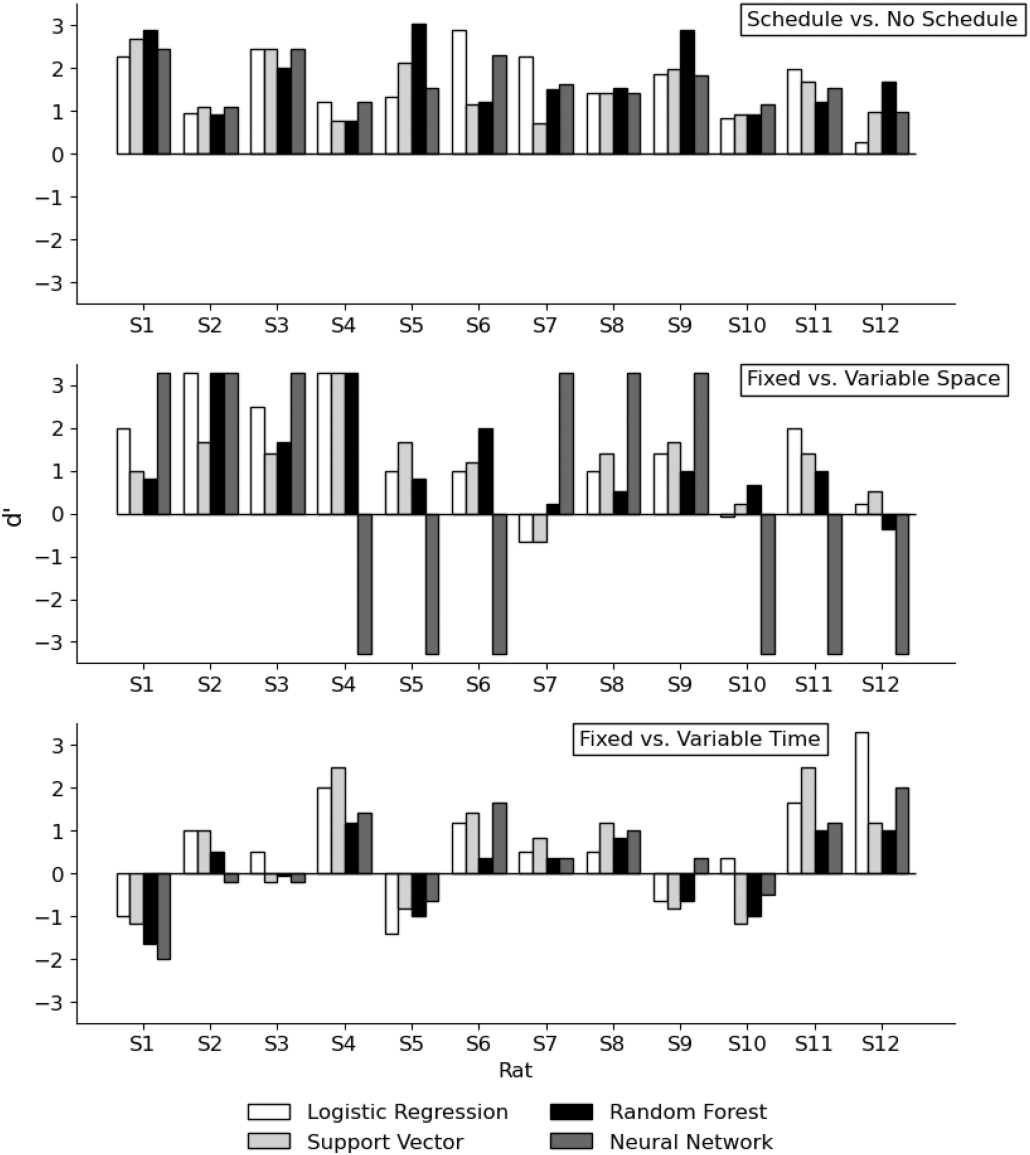
Discriminability indices (*d*′) of the models produced by the different algorithms for the three class labels.

Figure 2 presents the *d*′ of the models for each rat across the three class labels, and Table 3 presents the results of the Wilcoxon signed‐rank tests. The upper panel of Figure 2 shows that the models always produced positive *d*′ values when differentiating between the presence and absence of schedules, which resulted in all the Wilcoxon tests being statistically significant for this analysis. Table 4 includes the *c* values, sensitivity, and specificity for discriminating between the presence and absence of schedules. The logistic regression, the support vector classifier, and the artificial neural network did not produce systematic patterns in bias. However, the random forest procedure produced more negative bias and generally higher sensitivity than specificity, indicating that the models had a liberal bias (i.e., more likely to identify a schedule even when none was present). The confusion matrices that were used to compute these values for each rat and algorithm are available in the Supporting Information.

**TABLE 3:** Wilcoxon signed‐rank tests for each model (*α* = .004).

Model	*W*	*p*
Schedule vs. No schedule		
Logistic regression	0.0	<.001
Support vector classifier	0.0	<.001
Random forest	0.0	<.001
Artificial neural network	0.0	<.001
Fixed vs. Variable space		
Logistic regression	4.0	.003
Support vector classifier	3.0	.002
Random forest	2.0	.001
Artificial neural network	39.0	1.00
Fixed vs. Variable time		
Logistic regression	20.5	.176
Support vector classifier	22.0	.204
Random forest	35.0	.791
Artificial neural network	25.5	.339

**TABLE 4:** Discriminability indices (*d*′), *c* values, sensitivity, and specificity for the models identifying the presence or absence of a programmed schedule for each test rat.

	*d*′				*c*				Sensitivity				Specificity			
Rat	LR	SVC	RF	ANN	LR	SVC	RF	ANN	LR	SVC	RF	ANN	LR	SVC	RF	ANN
S1	2.28	2.69	2.90	2.46	0.14	−0.06	−0.61	0.05	0.84	0.92	1.00	0.88	1.00	1.00	0.80	1.00
S2	0.96	1.10	0.91	1.09	−0.23	−0.29	−1.29	−0.29	0.76	0.80	0.96	0.80	0.60	0.60	0.20	0.60
S3	2.46	2.46	2.00	2.46	0.05	−0.74	0.05	0.05	0.88	0.88	0.96	0.88	1.00	1.00	0.60	1.00
S4	1.21	0.77	0.77	1.21	−1.45	−1.67	−1.67	−1.45	1.00	1.00	1.00	1.00	0.20	0.00	0.00	0.20
S5	1.33	2.12	3.03	1.53	0.62	0.22	−0.23	0.51	0.52	0.80	0.96	0.60	1.00	1.00	1.00	1.00
S6	2.90	1.15	1.21	2.31	−0.61	−0.83	−1.45	−0.90	1.00	0.92	1.00	1.00	0.80	0.40	0.20	0.60
S7	2.28	0.72	1.50	1.64	0.14	−0.11	−1.00	0.46	0.84	0.68	0.96	0.64	1.00	0.60	0.40	1.00
S8	1.43	1.43	1.55	1.43	0.57	0.57	0.07	0.57	0.56	0.56	0.76	0.56	1.00	1.00	0.80	1.00
S9	1.86	1.99	2.90	1.84	0.35	0.29	−0.61	−0.08	0.72	0.76	1.00	0.84	1.0	1.0	0.80	0.80
S10	0.84	0.91	0.91	1.15	−0.16	−1.30	−1.30	−0.83	0.72	0.96	0.96	0.92	0.60	0.20	0.20	0.40
S11	1.99	1.68	1.21	1.55	0.28	0.00	−1.45	0.07	0.76	0.80	1.00	0.76	1.00	0.80	0.20	0.80
S12	0.26	0.99	1.68	0.99	0.71	0.35	0.00	0.35	0.28	0.56	0.80	0.56	0.80	0.80	0.80	0.80

*Note*: The training set involved 11 rats (all but the test rat). LR: logistic regression; SVC: support vector classifier; RF: random forest; ANN: artificial neural network.

The middle panel of Figure 2 shows that the models produced mostly positive *d*′ values when classifying fixed‐ and variable‐space conditions. A notable exception was the artificial neural network, which always predicted that the space dimension of the schedule was fixed. The classification of the artificial neural network was either always correct or always incorrect, which produced extreme *d*′ values. The Wilcoxon signed‐rank tests were significant for logistic regression, the support vector classifier, and random forest but not for the artificial neural network. The lower panel of Figure 2 presents the results for the discrimination of the FT and VT schedules. All models produced multiple *d*′ values of zero or less, suggesting that discrimination was limited. None of the Wilcoxon signed‐rank tests was statistically significant.

**FIGURE 3:**
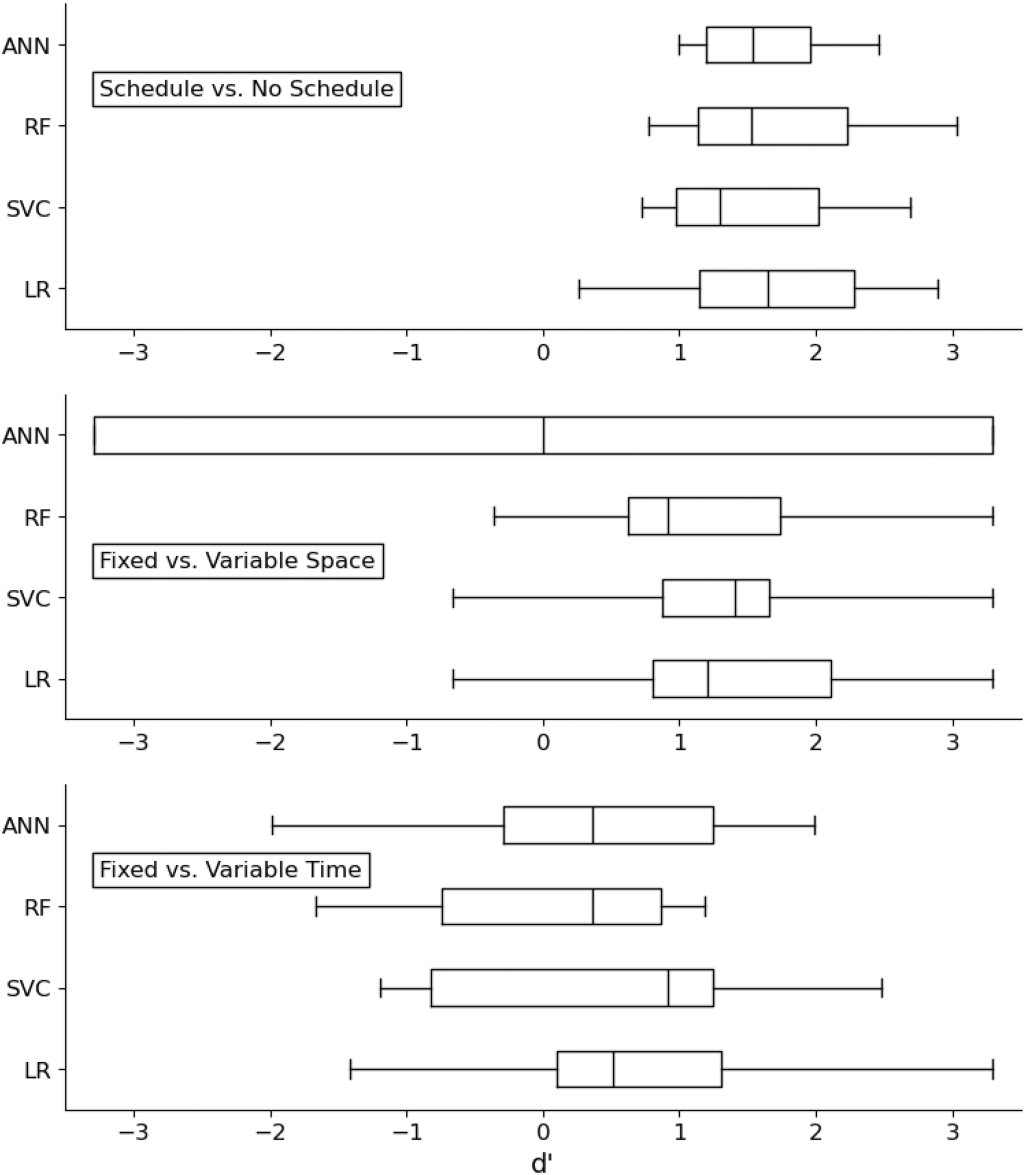
Box plot distributions of the discriminability indices (*d*′) of the models produced by the different algorithms for the three class labels. LR: logistic regression; SVC: support vector classifier; RF: random forest; ANN: artificial neural network.

To compare the performance of each algorithm on the three classes, Figure 3 presents box plots of the *d*′ values. The upper panel shows that each algorithm produced *d*′ values within the same range, which resulted in a Friedman chi‐square test that was nonsignificant, χ^2^(3) = 0.36, *p* = .95. In the middle panel, logistic regression, the support vector classifier, and random forest produced similar distributions. The artificial neural network produced a box plot that spans the whole range of *d*′ values. Nevertheless, the Friedman chi‐square test remained nonsignificant, χ^2^(3) = 0.51, *p* = .92. Finally, the lower panel shows that the boxes of each algorithm generally overlapped. This result is consistent with the Friedman chi‐square test, which was again nonsignificant, χ^2^(3) = 6.31, *p* = .10.

## DISCUSSION

Overall, our study shows that machine learning algorithms may accurately identify the presence or absence of programmed schedules as well as correctly differentiate between fixed‐ and variable‐space schedules in rats. Furthermore, logistic regression, support vector classifiers, random forests, and artificial neural networks generally produced similar classification when analyzing small sets of spatiotemporal data. To our knowledge, our study is the first to examine whether spatiotemporal data can identify the presence and the components of time‐based schedules in rats. The findings provide preliminary validation for the use of machine learning with spatiotemporal data to identify schedules as well as some information as to how the models could be improved in the future.

The main contribution of the current study is that we showed that researchers may use spatiotemporal data with machine learning to identify the presence or absence of schedules, which is a novel approach. One advantage of the proposed spatiotemporal measures is that they are not tied to a specific frame of reference, which could make it easier to adapt them to different environments. For example, researchers may measure traveled distance, location entropy, speed, and divergence regardless of the physical environment in which the rat is behaving. For their part, the dispenser entries could be replaced by sources of food and water, moving beyond the paradigm of focusing on lever presses (or key pecks in the case of pigeons). Moreover, detecting time‐based schedules appears to be important because many of the situations that are faced by living organisms remain noncontingent. For example, a rat foraging in the forest may only find food in specific locations on a schedule that is independent of its behavior. This study should be considered as a first step toward showing the feasibility and potential of relying on spatiotemporal data to identify reinforcement histories. More research must be conducted to improve the models and to examine their generalizability.

The study further extends the research literature by underlining the importance of comparing algorithms when addressing novel challenges with machine learning. Unexpectedly, the most advanced and complex algorithm (i.e., artificial neural network) did not produce models with higher discriminability than the simpler algorithms (e.g., logistic regression). One potential explanation for this observation is that the artificial neural network may have produced overfitting, reducing the accuracy of the models on the test data. This result supports the notion that more complex models are not necessarily better. Testing a simpler architecture (e.g., neural network with a single hidden layer) may have produced better generalizability. Additionally, the best algorithm may vary considerably based on the features and the class labels of the models. For example, Lanovaz and Hranchuk (2021) recently found that support vector classifiers were better than logistic regression at identifying single‐case graphs that showed a behavioral change. Thus, it is important that researchers compare algorithms when applying machine learning to new problems for the first time.

Another noteworthy observation is that our models failed to categorize FT and VT schedules correctly. There are at least three explanations for why the algorithms typically failed in this classification task. First, the FT and VT schedules were both signaled by a 3‐s tone prior to water availability. Thus, the spatiotemporal behavior of rats may not have differed considerably across the two conditions. An alternative explanation is that the space component of the schedule (i.e., fixed vs. variable space) may have interacted with the FT and VT schedules in such a way as to obscure the patterns of data for the algorithms. A third hypothesis is that the measures included in the features were not adequate to discriminate between FT and VT schedules. To examine this hypothesis, we developed models that included additional temporal features: the interresponse time as well as its standard deviation. Adding these features to our models did not significantly increase *d*′ (see Figure F in the Supporting Information). Researchers should thus examine other measures that could potentially better differentiate between the two types of (signaled) time‐based schedules.

Our results provide further support for the use of machine learning to identify the presence and some components of schedules of stimulus delivery in living organisms and extend those reported in prior research (e.g., Lusk, 2019; Plessas et al., 2022; Raphan et al., 2019). For example, the sensitivity and specificity of our most accurate models fall within the ranges reported by Plessas et al. (2022) on their test and generalization sets. However, all their models performed adequately, whereas ours did not. Several methodological differences between the two studies may explain these discrepancies. First, their model relied on data that were aggregated across 10 daily sessions, which may have increased the stability of responding. Therefore, each of their sample of 35 relied on 350 sessions. Our analysis relied on less than 10% of this amount of data, which may explain the lower values that we observed for some rats. One hypothesis is that these rats may have required more sessions to achieve stability in responding. Second, Plessas et al. tuned the hyperparameters of their model and presented the results of their best models. Because we did not have as much data, we could not tune the hyperparameters, which probably would have improved the models. Third, the nature of the input and output variables may also explain part of the differences. Responding on time‐based schedules may show more variability than responding on reinforcement schedules, which are contingent on behavior. Similarly, spatiotemporal data may also show more variability than key peck behavior, which would make the schedules more difficult to detect.

Our study has several limitations that should be considered when designing future experiments. The main limitation of our study is that we relied on a convenience sample, which had already been collected by the second and third authors. This convenience sample contained too few samples for us to examine the interactions between schedule components and to implement hyperparameter tuning. In an ideal data set, each rat would have been subjected to each of the four schedules for an equal number of sessions. The challenge was that we could not find any publicly available data set containing spatiotemporal data, so we relied on data that we had already collected from another experiment. Similarly, the phases were always conducted in the same order because it was important to test the hypothesis of the original study, but this could have produced order effects. Counterbalancing the order of the sessions appears to be important for isolating and detecting time‐based schedules more accurately in the future. Ultimately, researchers should collect more samples of different schedules while recording spatiotemporal data to provide more accurate and comprehensive models that can differentiate between different types of schedules.

## AUTHOR CONTRIBUTIONS

Marc J. Lanovaz: Conceptualization; formal analysis; methodology; software; writing – original draft preparation. Varsovia Hernandez: Investigation; methodology; resources; supervision; writing. Alejandro León: Conceptualization; data curation; investigation; methodology; resources; software; supervision; writing.

## CONFLICT OF INTEREST STATEMENT

The authors report no conflict of interest.

## ETHICS APPROVAL

All procedures described in the manuscript complied with university regulations for animal use and care, and with the Mexican norm NOM‐062‐ZOO‐1999 for Technical Specification for Production, Use and Care of Laboratory Animals.

## Supporting information


**Data S1:** Supporting Information

## Data Availability

All our data and code are freely available at https://osf.io/hsavx.

## Footnotes

^1^
As part of our preliminary analyses, we tested two additional sets of features: the first set included only the head entries into each dispenser, and the second set included only traveled distance, location entropy, standard deviation of speed, and divergence. Neither set of features produced adequate accuracy, which is why the models reported in the study integrated these eight features.

^2^
It should be noted that logistic regression is also used in traditional statistics. The difference between its use in traditional statistics and machine learning is methodological. In traditional statistics, researchers use logistic regression to identify statistically significant factors or predictors of the dependent variable by computing *p* values. In machine learning, researchers do not try to isolate the individual effect of the factors or predictors. The emphasis is on measuring and improving the accuracy of the model. Thus, the assumptions for using logistic regression in traditional statistics (e.g., multicollinearity) differ from those using logistic regression in machine learning.
