# Supplementary material for: Machine learning to detect schedules using spatiotemporal data of behavior: A proof of concept
Source: J Exp Anal Behav. 2025 Jun 30;124(1):e70029. doi: 10.1002/jeab.70029 (PMC12209495; doi:10.1002/jeab.70029)
Supplement: Supplementary file 1 — Data S1: Supporting Information [file JEAB-124-0-s001.pdf]

Supporting Information

Figure A

Confusion Matrix

	Detected	
Actual	No	Yes
No	True Negative	False Positive
Yes	False Negative	True Positive

Figure B

Confusion Matrices of Each Rat for the Logistic Regression

S1

	Detected	
Actual	No	Yes
No	5	0
Yes	4	21

S2

	Detected	
Actual	No	Yes
No	3	2
Yes	6	19

S3

	Detected	
Actual	No	Yes
No	5	0
Yes	3	22

S4

	Detected	
Actual	No	Yes
No	1	4
Yes	0	25

S5

	Detected	
Actual	No	Yes
No	5	0
Yes	12	13

S6

	Detected	
Actual	No	Yes
No	4	1
Yes	0	25

S7

	Detected	
Actual	No	Yes
No	5	0
Yes	4	21

S8

	Detected	
Actual	No	Yes
No	5	0
Yes	11	14

S9

	Detected	
Actual	No	Yes
No	5	0
Yes	7	18

S10

	Detected	
Actual	No	Yes
No	3	2
Yes	7	18

S11

	Detected	
Actual	No	Yes
No	5	0
Yes	6	19

S12

	Detected	
Actual	No	Yes
No	4	1
Yes	18	7

Figure C

Confusion Matrices of Each Rat for the Support Vector Classifier

S1

	Detected	
Actual	No	Yes
No	5	0
Yes	2	23

S2

	Detected	
Actual	No	Yes
No	3	2
Yes	5	20

S3

	Detected	
Actual	No	Yes
No	5	0
Yes	3	22

S4

	Detected	
Actual	No	Yes
No	0	5
Yes	0	25

S5

	Detected	
Actual	No	Yes
No	5	0
Yes	5	20

S6

	Detected	
Actual	No	Yes
No	2	3
Yes	2	23

S7

	Detected	
Actual	No	Yes
No	3	2
Yes	8	17

S8

	Detected	
Actual	No	Yes
No	5	0
Yes	11	14

S9

	Detected	
Actual	No	Yes
No	5	0
Yes	6	19

S10

	Detected	
Actual	No	Yes
No	1	4
Yes	1	24

S11

	Detected	
Actual	No	Yes
No	4	1
Yes	5	20

S12

	Detected	
Actual	No	Yes
No	4	1
Yes	11	14

Figure D

Confusion Matrices of Each Rat for the Random Forest

S1

	Detected	
Actual	No	Yes
No	4	1
Yes	0	25

S2

	Detected	
Actual	No	Yes
No	1	4
Yes	1	24

S3

	Detected	
Actual	No	Yes
No	3	2
Yes	1	24

S4

	Detected	
Actual	No	Yes
No	0	5
Yes	0	25

S5

	Detected	
Actual	No	Yes
No	5	0
Yes	1	24

S6

	Detected	
Actual	No	Yes
No	1	4
Yes	0	25

S7

	Detected	
Actual	No	Yes
No	2	3
Yes	1	24

S8

	Detected	
Actual	No	Yes
No	4	1
Yes	6	19

S9

	Detected	
Actual	No	Yes
No	4	1
Yes	0	25

S10

	Detected	
Actual	No	Yes
No	1	4
Yes	1	24

S11

	Detected	
Actual	No	Yes
No	1	4
Yes	0	25

S12

	Detected	
Actual	No	Yes
No	4	1
Yes	5	20

Figure E

Confusion Matrices of Each Rat for the Artificial Neural Network

S1

	Detected	
Actual	No	Yes
No	5	0
Yes	3	22

S2

	Detected	
Actual	No	Yes
No	3	2
Yes	5	20

S3

	Detected	
Actual	No	Yes
No	5	0
Yes	3	22

S4

	Detected	
Actual	No	Yes
No	1	4
Yes	0	25

S5

	Detected	
Actual	No	Yes
No	5	0
Yes	10	15

S6

	Detected	
Actual	No	Yes
No	3	2
Yes	0	25

S7

	Detected	
Actual	No	Yes
No	5	0
Yes	9	16

S8

	Detected	
Actual	No	Yes
No	5	0
Yes	11	14

S9

	Detected	
Actual	No	Yes
No	4	1
Yes	4	21

S10

	Detected	
Actual	No	Yes
No	2	3
Yes	2	23

S11

	Detected	
Actual	No	Yes
No	4	1
Yes	6	19

S12

	Detected	
Actual	No	Yes
No	4	1
Yes	11	14

Figure F

Results of the Models Produced by the Different Algorithms for the Fixed- vs. Variable-Time When the Mean and Standard Deviations of IRT Were Also Included as Features

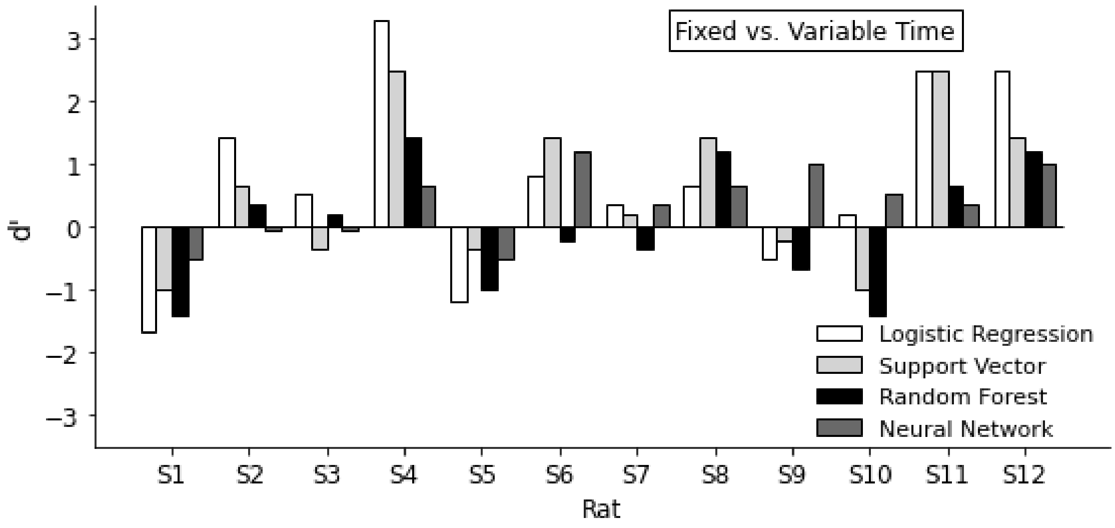

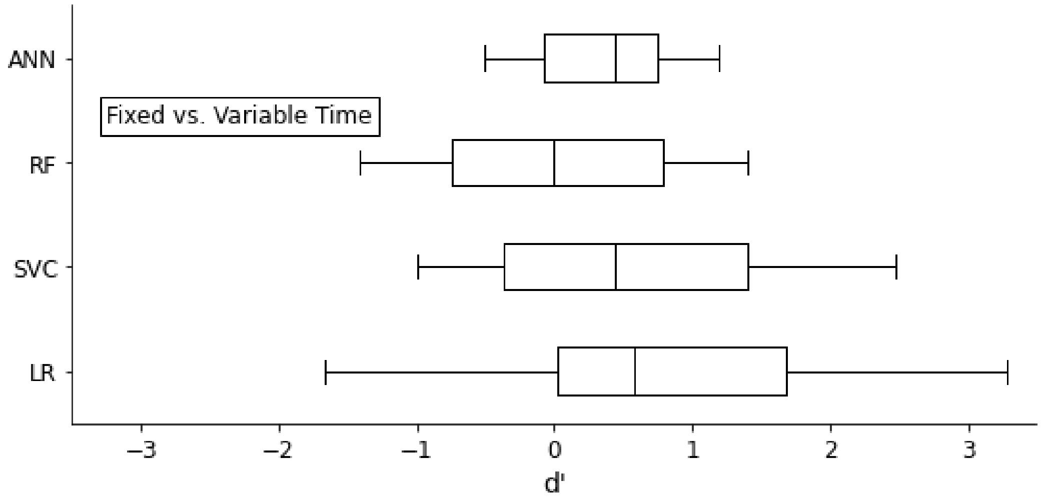

Wilcoxon Signed-Rank Test for Each Model

Model	*W*	*p*
Fixed vs. Variable Time		
Logistic Regression	19.5	.15
Support Vector machine	21.5	.20
Random Forest	38.5	.97
Artificial Neural Network	15.0	.06

Note. Friedman chi-square test: $\chi^2(3) = 5.51$, $p = .14$
